# Detection of silent myocardial ischemia in asymptomatic patients with diabetes: results of a randomized trial and meta-analysis assessing the effectiveness of systematic screening

**DOI:** 10.1186/1745-6215-12-23

**Published:** 2011-01-26

**Authors:** Michel M Lièvre, Philippe Moulin, Charles Thivolet, Michel Rodier, Vincent Rigalleau, Alfred Penfornis, Alain Pradignac, Michel Ovize

**Affiliations:** 1 Lyon 1 University, F-69003, UMR 5558, Louis Pradel Hospital, Bron F-69677 France; 2 Lyon 1 University, F-69003, INSERM, U870, Louis Pradel Hospital, Bron F-69677 France; 3 Lyon 1 University, INSERM, U870, IFR 62, Lyon, F-69008, Edouard Herriot Hospital, Lyon F-69008 France; 4 Carémeau Hospital, 30029 Nîmes Cedex, France; 5 Haut-Lévêque Hospital, Victor-Segalen-Bordeaux 2 University, Pessac 33600, France; 6 Jean Minjoz Hospital, 25030 Besançon Cedex, France; 7 Hautepierre University Hospital, F-67098, Strasbourg Cedex, France; 8 Lyon 1 University, F-69003 France, Louis Pradel Hospital, Bron F-69677, France

## Abstract

**Background:**

Most guidelines recommend a systematic screening of asymptomatic high risk patients with diabetes for silent ischemia, but the clinical benefit of this strategy has not been demonstrated compared with the simple control of cardiovascular risk factors. We sought to determine whether referring asymptomatic diabetic patients for screening of silent ischemia decreases the risk of cardiovascular events compared with usual care.

**Methods:**

DYNAMIT was a prospective, randomized, open, blinded end-point multicenter trial run between 2000 and 2005, with a 3.5 year mean follow-up in ambulatory care in 45 French hospitals. The study included 631 male and female with diabetes aged 63.9 ± 5.1 years, with no evidence of coronary artery disease and at least 2 additional cardiovascular risk factors, receiving appropriate medical treatment. The patients were randomized centrally to either screening for silent ischemia using a bicycle exercise test or Dipyridamole Single Photon Emission Computed Tomography (N = 316), or follow-up without screening (N = 315). The main study end point was time to death from all causes, non-fatal myocardial infarction, non-fatal stroke, or heart failure requiring hospitalization or emergency service intervention. The results of a meta-analysis of DYNAMIT and DIAD, a similar study, are also presented.

**Results:**

The study was discontinued prematurely because of difficulties in recruitment and a lower-than expected event rate. Follow-up was complete for 98.9% patients regarding mortality and for 97.5% regarding the main study end point. Silent ischemia detection procedure was positive or uncertain in 68 (21.5%) patients of the screening group. There was no significant difference between the screening and the usual care group for the main outcome (hazard ratio = 1.00 95%CI 0.59 to 1.71). The meta-analysis of these and DIAD results gave similar results, with narrower confidence intervals for each endpoint.

**Conclusions:**

These results suggest that the systematic detection of silent ischemia in high-risk asymptomatic patients with diabetes is unlikely to provide any major benefit on hard outcomes in patients whose cardiovascular risk is controlled by an optimal medical treatment.

**Trial registration:**

ClinicalTrials.gov: NCT00627783

## Background

Cardiovascular diseases and particularly coronary heart disease (CHD) are the leading causes of death in patients with type-2 diabetes [1,2]. CHD is often asymptomatic in these patients, and is therefore at an advanced stage when it becomes clinically manifest [3,4]. Consequently, most guidelines recommend a systematic screening by stress testing of asymptomatic high risk patients with diabetes [5,6]. Systematic screening is however costly, and has been shown to have a rather low yield [3,7-9]. Moreover, percutaneous coronary intervention (PCI) has not been shown superior to conservative therapy in non-acute CHD [10], whereas control of cardiovascular risk factors has been shown beneficial in diabetic patients. Consequently, the paradigm of a more aggressive management of patients with silent ischemia is unlikely to translate into obvious benefits. We therefore decided to set up a multicenter, double-blind randomized, unblinded strategy trial: DYNAMIT (Do You Need to Assess Myocardial Ischemia in Type-2 diabetes) comparing systematic referral to a cardiologist for the assessment of silent myocardial ischemia with usual medical care. We also present a meta-analysis including the results of DYNAMIT and those of DIAD, a similar American study that was run during the same period, and published in 2009 in JAMA.

## Methods

### Patients

The study was approved by the Lyons investigational review board and authorized by the French ministry of health. Ambulatory patients who consulted a diabetes specialist in a hospital were eligible if they were 55 to 75 years old, had type-2 diabetes and at least two of the following cardiovascular risk factors: urinary albumin excretion > 30 mg/L or > 30 mg/24 hours, treated or untreated hypertension, treated or untreated lipid abnormality, peripheral arterial disease, history of transient ischemic accident, tobacco consumption and familial history of premature cardiovascular disease. Patients were excluded if they had a history of myocardial infarction, coronary artery disease, or stroke, a previous positive stress test or myocardial perfusion imaging, or a negative stress test or myocardial perfusion imaging within the last three years. Patients gave a written informed consent and provided the coordinating centre with their full name, address and phone number, and those of at least one relative.

### Procedures

Specialists in diabetes care in 45 French hospitals randomized the eligible patients to the screening or usual care group by using a central, automated telephone procedure stratified by investigating centre. In the usual care group, patients were treated according to current guidelines but were not referred to a cardiologist. Patients assigned to the screening group were referred to a cardiologist for a systematic detection of silent ischemia by a bicycle exercise test performed according to the French Society of Cardiology protocol [11] after washout of cardiovascular medications likely to interfere with the test. Dipyridamole Single Photon Emission Computed Tomography (SPECT) was used in patients unable to perform the exercise test, with a sub-maximal negative exercise test result or with electrocardiographic abnormalities impairing the interpretation of the exercise test. SPECT results showing small defects (uncertain results) were grouped with definitely abnormal results (positive stress test or medium to large SPECT perfusion defects), due to the small number of patients. Subsequent investigations (such as coronary angiography) and treatments (such as revascularization procedures) were left at the cardiologist's decision.

### Follow-up

Baseline cardiovascular history and risk factors, results of the cardiac investigations, cardiovascular and anti-diabetic medications at discharge were entered in the case report form. Patients were followed-up after discharge by the coordinating centre until the end of the study. Patients were asked by mail every six months if they had been hospitalized since the last contact. Patients who did not answer were contacted by telephone. The study investigators were asked at the end of the follow-up to complete the information given by the patients. In case of hospitalization for identified or possible cardiovascular causes, contact was established with the hospital and with the patient's primary care physician to document the event. Follow-up was completed by an administrative survival inquiry. At the occasion of the last contact, patients were asked to give a list of all the medications that they used daily.

### End points

The main end point was time to death from all causes, non-fatal myocardial infarction (MI), non-fatal stroke, or heart failure requiring hospitalization or emergency service intervention. Secondary end points included the components of the main end point, unstable angina requiring hospitalization, coronary events (fatal or non-fatal MI, hospitalized unstable angina, or heart failure requiring hospitalization or emergency service intervention), and coronary revascularization (angioplasty or coronary artery bypass graft, including procedures decided after the first silent ischemia detection procedure in the screening group). All study outcomes were adjudicated by a blinded independent event committee.

### Statistical analysis

It was expected that 22% patients of the control group would experience a main study end point over 4 years of follow-up (5.5% per year). Three thousand patients were needed to obtain an 80% power to detect a 20% relative reduction in the risk of main end point in the screening compared with the control group.

The analysis was on intention to treat. All reported levels of significance are two-sided, and 95% confidence intervals (95%CI) are reported where appropriate. Time-to-event curves were constructed according to the Kaplan-Meier method. The study groups were compared for the main end point and coronary events by the means of a Cox proportional model adjusted on age, sex and presence of albuminuria. Hazard ratios (HR) are presented with their 95% confidence interval. Other variables were compared with the use of chi-square or t-tests as appropriate.

### Meta-analysis

An electronic search for reports of trials similar to DYNAMIT was performed using the PubMed database and the key words "("ischemia"[All Fields] OR "ischemia"[MeSH Terms] OR "ischemia"[All Fields]) AND asymptomatic[All Fields] AND (("diabetes mellitus"[MeSH Terms] OR ("diabetes"[All Fields] AND "mellitus"[All Fields]) OR "diabetes mellitus"[All Fields] OR "diabetes"[All Fields] OR diabetic[All Fields]) AND Randomized Controlled Trial[ptyp]". The selection criteria for including a trial in the meta-analysis were the following: randomized trial comparing screening with stress test or SPECT with no screening in asymptomatic patients with diabetes followed-up for at least one year, and availability of cardiovascular events.

Relative risks of events were computed and combined using the EasyMA software [12,13]. For each comparison, the result is given as the relative risk and its 95% confidence interval (95%CI). Statistical significance of the difference is given by the association p-value. Heterogeneity between trials and between sub-groups was tested by the Cochrane Q-test.

### Role of the funding source

The sponsors of the study had no role in study design, data collection, data analysis, data interpretation, or writing of the report. The corresponding author (ML) had full access to all the data in the study. The DYNAMIT group had final responsibility for the decision to submit for publication.

## Results

### Study course

**Figure 1:**
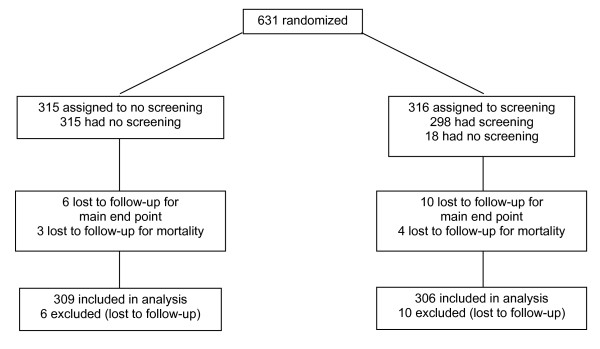
**Study flow-chart**.

Recruitment began in December 2000. Mid-June 2003, only 628 patients had been randomized by 45 centers, and 30 main end points had been observed in 18 participants. Consequently, considering the irremediable lack of power the study steering committee decided to stop recruitment on July 11, 2003. At this time, 631 randomizations had been recorded, 316 in the screening and 315 in the control group. Follow-up continued until July 30, 2005 (Figure 1).

Follow-up was complete for 624 patients (98.9%) regarding mortality and for 615 (97.5%) regarding the main study end point. Main follow-up duration was 3.5 years in both groups.

### Patient characteristics

Study groups were well matched at baseline regarding patient characteristics and cardiovascular risk factors (Table 1). Body mass index and glycated hemoglobin concentrations were increased as expected in this population of patients referred to tertiary care centre. Few patients had a history of peripheral arterial disease, transient ischemic attack or heart failure.

**Table 1:** Baseline patient characteristics by randomization group.

Parameter	Usual care	Screening
Age (years)	63.7 ± 6.4	64.1 ± 6.4

Males (%)	53.7	55.4

BMI (kg/m²)	30.8 ± 5.3	30.4 ± 4.7

HbA1C (%)	8.7 ± 2.0	8.6 ± 2.2

Lipid abnormality (%)	81.6	80.4

History of TIA (%)	3.8	5.4

History of heart failure (%)	0	1.0

History of peripheral arterial disease (%)	14.3	13.9

Familial history of cardiovascular disease (%)	21.0	21.8

Tobacco consumption (%)	14.6	17.4

Hypertension (%)	88.3	89.2

Increased urinary albumin excretion (%)	40.3	44.0

BMI = body mass index; TIA = transient ischemic attack. Quantitative values are given as means and (SD)

### Stress test results and treatment at hospital discharge

In the screening group, 231 (73.1%) patients underwent an exercise test and 98 (31.0%) SPECT. The result of this first step of the silent ischemia detection procedure was definitely abnormal or uncertain in 68 (21.5%) patients. Coronary angiography was performed in 38 (12% patients). Subsequent coronary angioplasty was performed in 9 patients, among who 7 received at least one stent, and 3 had coronary artery bypass graft.

Medical treatments at hospital discharge following the first study visit were similar, with only a trend toward a more frequent use of antiplatelet drugs in the screening group (Table 2). Reflecting the recommendations in use at the time of the study inception, statins were used by only one third of patients, but most of the patients (70%) were treated by drugs interfering with the renin-angiotensin system. The use of insulin in 45% patients suggests an advanced state of metabolic impairment.

**Table 2:** Treatments at hospital discharge (% patients) by randomization group

Medication	Usual care	Screening
Metformin	67.9	61.7

Sulfonylurea	52.4	47.5

Other oral antidiabetic drugs	23.5	25.6

Insulin	44.4	45.6

Statin	35.9	33.5

Fibrate	24.1	25.3

Aspirin	24.1	29.1

Other antiplatelet drug	5.4	7.6

Diuretic	37.8	42.7

Beta-blocker	16.5	23.4

ACE-inhibitor	53.0	50.6

Angiotensin receptor blocker	15.9	19.0

Calcium channel blocker	27.6	25.6

### Outcomes

**Figure 2:**
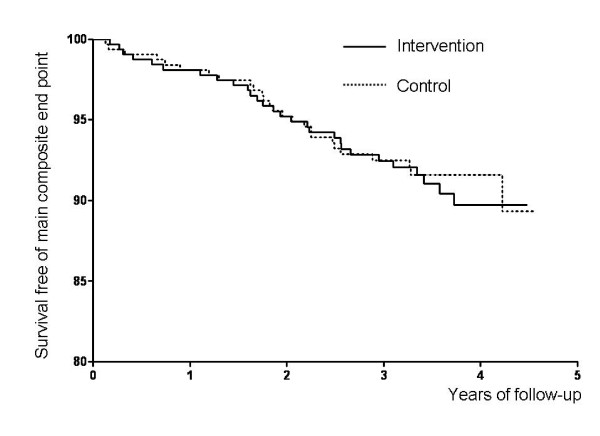
**Survival curve for the main composite end-point (time to death from all causes, non-fatal myocardial infarction, non-fatal stroke, or heart failure requiring hospitalization or emergency service intervention)**.

Twenty-eight patients experienced at least one primary end point in the screening group and 26 in the control group (Figure 2), with no significant difference between the groups (Adjusted HR = 1.00; 95%CI 0.59 to 1.71). The only adjustment parameter that had a significant prognostic value was increased urinary albumin excretion (HR = 2.58; 95%CI 1.45 to 4.58). There was a trend toward a lower number of MIs and a higher number of strokes in the screening compared with the control group (Table 3), but the absolute numbers of events were small. Coronary events occurred in 13 and 15 patients in the screening and control groups respectively (HR = 0.77; 95%CI 0.37 to 1.63). Coronary revascularization was performed in 18 and 21 patients in the screening and control groups respectively (p = 0.61). Overall the absolute risk of major cardiovascular event was 2.4% per year.

**Table 3:** Adjudicated events (number of patients with at least one event during follow-up) by randomization group.

	Usual care	Screening
Main end point *	26	28

Myocardial infarction	8	4

Stroke	4	9

Hospitalized cardiac failure	4	5

All cause deaths	13	15

Coronary events †	15	13

Revascularization	21	18

* death from all causes, non-fatal myocardial infarction, non-fatal stroke, or heart failure requiring hospitalization or emergency service intervention. †fatal or non-fatal MI, hospitalized unstable angina, or heart failure requiring hospitalization or emergency service intervention.

### Safety

No serious adverse event was reported during the initial hospitalization of patients. Adverse events that occurred during follow-up were not recorded.

### Meta-analysis

Our PubMed search yielded only one eligible trial, the Detection of Ischemia in Asymptomatic Diabetics (DIAD) [14]. This study was launched in 2000 and included 1123 50-75 years old patients with diabetes, 561 of which were allocated to SPECT and 562 to follow-up only. Any evidence of coronary heart disease, and stress test or coronary angiography within the prior 3 years were exclusion criteria. The prevalence of SPECT abnormalities (including small defects) was the same as that of silent ischemia in our study (22%) whether the patients had or not at least two additional cardiovascular risk factors. With a longer follow-up duration of 4.8 years, the yearly risks of death from all causes (0.62%) and of cardiac death or non-fatal MI (0.59%) were much smaller than in DYNAMIT (respectively 1.27% and 0.95%).

**Figure 3:**
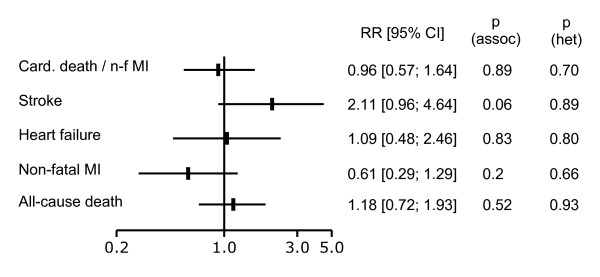
**Meta-analysis of the DYNAMIT and DIAD study results**. Relative risks (RR) and 95% confidence intervals (95% CI), with usual care as the reference group. Main outcome of the DIAD study (cardiac death or non-fatal myocardial infarction (Card. Death/n-f MI) and other outcomes. P(assoc): association p-value. P(het): heterogeneity p-value.

The results of the meta-analysis are shown in Figure 3. Unfortunately, we were unable to obtain the DIAD results corresponding to our main end point from the DIAD investigators. The main DIAD end point (cardiac death or non-fatal MI) could however be obtained for DYNAMIT, allowing its inclusion in the meta-analysis. No heterogeneity was detected between the trials (p > 0.7 for all end points). No significant favorable effect of screening was detected, although there was a favorable trend for non-fatal MI (RR = 0.61 [0.29 to 1.29], p = 0.20), and an adverse trend for stroke (RR = 2.11 [0.96 to 4.64], p = 0.06).

## Conclusions

Compared with no cardiologic investigation, the systematic detection of silent ischemia in asymptomatic patients with type-2 diabetes at a high cardiovascular risk did not result in a lower incidence of death, non-fatal MI, non-fatal stroke, or heart failure requiring hospitalization over 3.5 years of follow-up in this randomized strategy trial. No significant difference either was detected regarding the incidence of coronary events and revascularization. Due to premature study interruption following difficulties in patient recruitment, the trial had an insufficient power and does not allow ruling out a 41% decrease, as well as a 71% increase in the risk of primary end point in the screening group. However, a meta-analysis of DYNAMIT and DIAD, a very similar, slightly more powerful study, gave the same results, narrowing confidence intervals for all end points. It is unfortunate we could not obtain the DIAD results for our composite end point from the trial investigators. Considering all the components of this end point in the DIAD study, it is evident, however, that the results would have been very close to those of DYNAMIT. Owing to the number of secondary end points tested, and the fact that strokes were not related to study procedures, the adverse trend regarding stroke is likely to be a chance finding.

DYNAMIT has several weaknesses that cannot be ignored. Firstly, it was an open trial, like all strategy trials, and so was DIAD. A selection bias due to the absence of blinding is however unlikely because the central randomization procedure recorded the patient identity before giving the randomization group. Additionally the investigator therapeutic choices might have been influenced by the group to which the patient belonged. In fact, this bias is unlikely since treatments prescribed to the patients at hospital discharge were very similar in both groups. The insufficient statistical power is a major drawback of DYNAMIT and DIAD. Among the multiple reasons that led to recruitment difficulties and early termination of our trial, the most important was the existence of professional recommendations to detect silent ischemia in patients eligible to the trial, which limited the number of investigators and resulted in a high proportion of diabetic patients already screened by an exercise test in the three preceding years. This may also explain why the recruited patients had a lower-than-expected risk of primary events despite a conservative inclusion of patients with 2 additional risk factors. Moreover DYNAMIT was an academic trial and recruitment was hampered by competition with industry-sponsored trials targeting the same population. It is of note that DIAD, despite its bigger sample size and longer follow-up, was only slightly more powerful than DYNAMIT because it included patients at a much lower risk of events. In fact, only 60% of the DIAD patients had at least 2 cardiovascular risk factors compared with 100% in DYNAMIT.

One of the salient features of DYNAMIT was the centralized independent follow-up. Only a small number of patients were lost to follow-up over 3.5 years although the protocol did not require patients to come back to refill a prescription or have a test done. Contact with patients was excellent, and those who were lost to follow-up had moved without notifying the investigator, their primary physician, and even their relatives. These patients would have been missed by a classical follow-up as well.

The DYNAMIT and DIAD studies bring thoughtful information. They are to our knowledge the only randomized trials to compare systematic silent ischemia detection with follow-up only in asymptomatic diabetic patients with additional cardiovascular risk factors. Although both studies lacked power, they did not suggest that systematic screening for silent myocardial ischemia in this type of patients allowed making better therapeutic options than watchful follow-up.

Our findings must be considered in view of the COURAGE and BARI 2D trial results [15,16]. In Courage, 2287 patients with objective evidence of myocardial ischemia were randomized to PCI and optimal medical therapy versus medical therapy alone. After 4.6 years, there was no difference between the study groups in the composite of death or MI, and death, MI or stroke. Similar results were obtained in the 766 patients with diabetes (hazard ratio = 0.99; 95%CI 0.73 to 1.32). In BARI 2D, 2368 patients with both type 2 diabetes and heart disease were randomized to either prompt revascularization with intensive medical therapy or intensive medical therapy alone. At 5 years, there was no difference between the groups for survival (revascularization 88.3%, medical therapy 87.8%, p = 0.97), nor for MI or stroke-free survival (77.2% vs 75.4%, p = 0.70). Thus, systematic revascularization does not appear to improve the prognosis of type-2 diabetics with coronary heart disease when metabolic and cardiovascular risk factors are controlled appropriately. Consequently, increasing the access of asymptomatic patients with diabetes to PCI through the detection of silent ischemia cannot be expected to improve prognosis.

Systematic screening of asymptomatic patients with diabetes is costly, and may affect negatively the quality of life of one in five patients by putting them into the heart disease population, with no expectable benefit. On the other hand, the majority of patients, who will test negative, may be falsely reassured, and less likely to adhere to efficient medical treatment and healthy lifestyle. Diabetic asymptomatic patients who may benefit from screening are still to be defined.

## Competing interests

The authors declare that they have no competing interests.

## Authors' contributions

M Lièvre was a member of the steering committee and responsible for the coordination, data management and analysis of the study. He was the main writer of the article and had full access to all of the data in the study and takes responsibility for the integrity of the data and the accuracy of the data analysis.

P Moulin was a member of the steering committee, an investigator, and participated in the writing of the article.

C Thivolet was a member of the steering committee, an investigator, and participated in the writing of the article.

M Rodier was a member of the steering committee and an investigator.

V Rigalleau, F Penfornis, and A Pradignac were investigators.

M Ovize was the main investigator and a member of the steering committee. He participated in the writing of the article.

All authors read and approved the final manuscript.
